# Whole Genome Sequence Analysis of *Listeria monocytogenes* Isolates Obtained from the Beef Production Chain in Gauteng Province, South Africa

**DOI:** 10.3390/microorganisms12051003

**Published:** 2024-05-16

**Authors:** James Gana, Nomakorinte Gcebe, Rian Edward Pierneef, Yi Chen, Rebone Moerane, Abiodun Adewale Adesiyun

**Affiliations:** 1 Department of Production Animal Studies, Faculty of Veterinary Science, University of Pretoria, Private Bag X04, Onderstepoort, Pretoria 0110, South Africa; jamesgana38@gmail.com (J.G.); rebone.moerane@up.ac.za (R.M.); 2 Department of Agricultural Education, Federal College of Education, Kontagora 923101, Niger State, Nigeria; 3 Bacteriology Department, Onderstepoort Veterinary Research, Agricultural Research Council, Pretoria 0110, South Africa; gceben@arc.agric.za; 4 Department of Biochemistry, Genetics and Microbiology, University of Pretoria, Pretoria 0001, South Africa; rian.pierneef@up.ac.za; 5 Centre for Bioinformatics and Computational Biology, University of Pretoria, Pretoria 0001, South Africa; 6 Microbiome@UP, Department of Biochemistry, Genetics and Microbiology, University of Pretoria, Pretoria 0001, South Africa; 7 Center for Food Safety and Applied Nutrition, US Food and Drug Administration, 5001 Campus Dr. Room 4E-007/Mailstop HFS-710, College Park, MD 20740, USA; yi.chen@fda.hhs.gov; 8 School of Veterinary Medicine, Faculty of Medical Sciences, University of the West Indies, St. Augustine 685509, Trinidad and Tobago

**Keywords:** beef production chain, *Listeria monocytogenes*, whole-genome sequencing, sequence type, clonal complexes, virulence factor, antimicrobial resistance genes, plasmids, South Africa

## Abstract

The study used whole-genome sequencing (WGS) and bioinformatics analysis for the genomic characterization of 60 isolates of *Listeria monocytogenes* obtained from the beef production chain (cattle farms, abattoirs, and retail outlets) in Gauteng province, South Africa. The sequence types (STs), clonal complexes (CCs), and the lineages of the isolates were determined using in silico multilocus sequence typing (MLST). We used BLAST-based analyses to identify virulence and antimicrobial genes, plasmids, proviruses/prophages, and the CRISPR-Cas system. The study investigated any association of the detected genes to the origin in the beef production chain of the *L. monocytogenes* isolates. Overall, in 60 isolates of *Listeria monocytogenes*, there were seven STs, six CCs, forty-four putative virulence factors, two resistance genes, one plasmid with AMR genes, and three with conjugative genes, one CRISPR gene, and all 60 isolates were positive for proviruses/prophages. Among the seven STs detected, ST204 (46.7%) and ST2 (21.7%) were the most prominent, with ST frequency varying significantly (*p* < 0.001). The predominant CC detected were CC2 (21.7%) and CC204 (46.7%) in lineages I and II, respectively. Of the 44 virulence factors detected, 26 (across *Listeria* Pathogenicity Islands, LIPIs) were present in all the isolates. The difference in the detection frequency varied significantly (*p* < 0.001). The two AMR genes (*fosX* and *vga*(*G*)) detected were present in all 60 (100%) isolates of *L. monocytogenes.* The only plasmid, NF033156, was present in three (5%) isolates. A CRISPR-Cas system was detected in six (10%), and all the isolates carried proviruses/prophages. The source and sample type significantly affected the frequencies of STs and virulence factors in the isolates of *L. monocytogenes.* The presence of *fosX* and *vga*(*G*) genes in all *L. monocytogenes* isolates obtained from the three industries of the beef production chain can potentially cause therapeutic implications. Our study, which characterized *L. monocytogenes* recovered from the three levels in the beef production chain, is the first time genomics was performed on this type of data set in the country, and this provides insights into the health implications of *Listeria.*

## 1. Introduction

*Listeria monocytogenes* is the primary cause of human cases and listeriosis outbreaks, and has a considerable negative economic impact on society and the food industry [1]. Although *L. monocytogenes* is the only recognized human pathogen among *Listeria* species, it is also pathogenic for animals [2,3]. *L. ivanovii* is the only other pathogen responsible primarily for listeriosis in animals [4], but it has been reported to cause listeriosis in humans [5].

Human listeriosis outbreaks have been documented globally, including the world’s largest outbreak reported in South Africa [3,6]. *L. monocytogenes* has been implicated in sporadic cases and listeriosis outbreaks, some of which may be multi-country, and the definitive source may be unknown [7]. The European Food Safety Authority [8] reported 2183 confirmed invasive human cases of *L. monocytogenes* in 2021, and the case fatality rate was high (13.7%), similar to 2020 [8], confirming listeriosis as one of the most severe foodborne diseases.

*Listeria monocytogenes* is an important foodborne zoonotic agent, and it has been demonstrated to be present in several food types and, therefore, poses a food safety risk [9]. Meat and meat products are part of the daily human diet because they contain high nutritional value, including proteins, important amino acids, vitamins, and minerals [10]. The dietary components in meat function as ‘natural media’ for microorganisms such as *L. monocytogenes* [11]. It has been documented that consuming ready-to-eat (RTE) meat products has been responsible for approximately 30% of human listeriosis outbreaks between 2008 and 2015 in Belgium [12]. Thus, contaminated RTE meat products are a significant concern for public health [13]. Unlike other foodborne pathogens, *L. monocytogenes* can survive harsh food processing environments such as acidic pH, low moisture content or water activity, and refrigeration temperatures, thus facilitating its proliferation in the food environment [14]. Due to the pathogen’s ubiquity, contamination of meat and meat products occurs at various processes, including RTE products [15] and distribution stages [16].

For decades, the traditional serotyping of *L. monocytogenes* has been used to characterize isolates recovered from several sources for investigative purposes [17]. However, researchers and diagnosticians now rely on more sensitive, specific, and accurate molecular methods to diagnose, confirm, and characterize *L. monocytogenes* isolates. Some of these methods include the polymerase chain reaction (PCR), multi-virulence-locus sequence typing (MVLST), multi-locus sequence typing (MLST), pulse-field gel electrophoresis (PFGE), and whole-genome sequencing (WGS), which are now being used [3,18,19].

The sequence types (STs) and the clonal complexes (CC) of *L. monocytogenes* have been used to characterize the pathogen [20,21], and numerous STs have been identified in *L. monocytogenes* isolates worldwide [22]. Of significance is the frequent association of some STs and CCs with isolates of *L. monocytogenes* that are implicated with human listeriosis, thus making the detection of these STs and CCs critical in epidemiological investigations [21,23,24,25,26].

The virulence factors possessed by strains of *L. monocytogenes* have been associated with their pathogenicity, especially those present in the *Listeria* Pathogenicity Islands (LIPIs) [3,27,28]. The virulence factors in the LIPIs play vital roles in the pathogenicity of *L. monocytogenes*. It has been demonstrated that the virulence genes in the LIPI-1 and LIPI-3 clusters play a role in the infectious life cycle and survival in the food processing environment [28]. *Listeria monocytogenes* also possesses several other virulence factors, including internalins, listeriolysin O, and listeriolysin S, which play an important regulatory role in its pathogenicity [29,30].

Antimicrobial resistance (AMR) genes have been documented in *L. monocytogenes* isolates, and are produced to facilitate the development of phenotypic resistance to antimicrobial agents [31]. The frequencies of AMR genes in *L. monocytogenes* have been reported to vary in isolates recovered from cattle farms, abattoirs, and retail outlets [32,33,34]. The abuse and overuse of antimicrobial agents in human and animal populations result in developing resistance to antimicrobial agents, which is facilitated by the production of appropriate resistance genes as an adaptive response by the pathogen [35,36]. Wiktorczyk-Kapischk et al. [36] have reported that the horizontal gene transfer (HGT) of mobile genetic elements, including plasmids, transposons-carrying resistant genes, and the activation of efflux pump systems are primarily responsible for the resistance of *L. monocytogenes* to antimicrobial agents.

Plasmids are found in several bacterial pathogens, including *Listeria* spp. [37], and of significance is their ability to carry genetic materials with the potential to encode AMR [38]. In addition, some plasmids provide other benefits to the host cells with potential contribution to stress survival [39]. Some of the plasmids detected in *L. monocytogenes* include plasmid profiles (N1-011A, J1776, and pLM5578), which were detected in *L. monocytogenes* isolates recovered from food processing environments in South Africa [40].

Prophages/proviruses are commonly found in *Listeria* genomes and have been reported to play an essential role in bacterial evolution, survival, and persistence [41]. They are also known to mediate defence against phage infection through diverse mechanisms in bacteria [42]. Several frequencies and types of prophages have been reported in *L. monocytogenes* from many sources [40,43,44,45].

CRISPR-Cas system exists in several bacteria, including *Listeria* spp., which acts as an adaptive immune system of bacteria and is known to help invade the host immune system [46]. Several types of CRISPR-Cas have been reported in *Listeria* spp., which include Cas-type IA, Cas-type IB, and Cas-type IIA [47]. Various CRISPR-Cas systems in *L. monocytogenes* isolates recovered from cattle farms, abattoirs, foods, food processing environments, and retail outlets have been found [46,48,49,50].

South Africa experienced a large outbreak of human listeriosis in 2017–2018 [6] caused by a strain of *L. monocytogenes*, ST6, due to the consumption of ‘polony’, an RTE pork product [51]. Earlier reports in the country have documented the occurrence of listeriosis in livestock [52]. Reports exist using WGS to characterize *L. monocytogenes* recovered from the large human listeriosis outbreak [24], isolates of *Listeria* spp. obtained from beef processing environments [40] and the red meat and poultry value chain [53]. Most recently, Gana et al. [54] characterized the *L. innocua* isolates recovered from cattle farms, abattoirs, and retail outlets using WGS. To date, there are limited data on the WGS analysis of the occurrence of *L. monocytogenes* strains in the beef production chain’s three levels (production, processing, and retailing) in Gauteng province, South Africa.

Therefore, the current study was conducted to characterize the *L. monocytogenes* molecularly isolates from the three levels (cattle farms, abattoirs, and retail outlets) of the beef production chain in Gauteng province using WGS. The study also determined the occurrences of sequence types, virulence factors, AMR genes, plasmids, provirus/prophage, and CRISPR-Cas and the potential effects of the origin of *L. monocytogenes* isolates (sources and sample/food types) on their profiles.

## 2. Materials and Methods

### 2.1. Origin of the Isolates of L. monocytogenes Used in Our Study

Sixty isolates of *L. monocytogenes*, on which WGS and bioinformatics analyses were performed in the current study, were recovered from cattle farms, cattle abattoirs, and retail outlets in Gauteng province. An earlier study [55] provided a detailed description of the isolates regarding the sources and types of samples processed.

### 2.2. Study Design and Sources of Samples

The study’s design was to conduct three cross-sectional studies, one each on cattle farms (production), cattle abattoirs (processing), and retail outlets (retailing), which constitute the three industries in the beef production system in Gauteng province. The sample size used in each industry was determined using the formula recommended by Thrusfield [56]. Our earlier report on *L. innocua* recovered from the same samples used in the current study has provided a flow chart for the relevant sampling to each industry [54].

### 2.3. Investigation of the Variables or Factors Associated with the Distribution of Genomic Characteristics of L. monocytosis Isolates

Details of the variables investigated in the current study were provided in our earlier study [54]. Briefly, the variables investigated included the type of farm (communal, cow-calf, and feedlot) and feed (grass, grain, and silage). The size (butcheries, high throughput, and low throughput) and practices (pre- and post-evisceration) were investigated as variables at abattoirs. The effects of the size (chain, large, medium, and small) and the types of beef and beef products retailed (raw and processed beef products, including RTE products) were the variables investigated at the retail outlets.

### 2.4. Isolation and Identification of L. monocytogenes

The *L. monocytogenes* isolates stored at −80 °C were confirmed using standard bacteriological and molecular (multiplex PCR) techniques [54,55]. There were 60 isolates of *L. monocytogenes*, comprising 11, 12, and 37 isolates originating from cattle farms, cattle abattoirs, and retail outlets, respectively, studied.

### 2.5. DNA Extraction from L. monocytogenes Isolates

Our study extracted DNA from the 60 isolates of *L. monocytogenes* using the Qiagen DNAEasy Blood & Tissue kit (Thermo Fisher Scientific, Johannesburg, South Africa), manual, Gram-positive protocol, according to the manufacturer’s instructions.

### 2.6. Whole-Genome Sequencing, Genomic Analysis, Assembly, and Annotation

An Illumina MiSeq platform (250-bp paired-end reads; Illumina, Inc., San Diego, CA, USA) was used to sequence all isolates of *L. monocytogenes* with the Nextera XT library preparation kit per the manufacturer’s instructions.

Quality control, including adapter removal, was conducted using BBDuk (v.38.91; https://jgi.doe.gov/data-and-tools/software-tools/bbtools/bb-tools-user-guide/bbduk-guide/) (accessed on 6 September 2022). SPAdes v.3.15.3 [57] created a de novo assembly of each isolate with only contigs longer than 500 bp retained for further analysis. We assessed the completeness and contamination of the assemblies with CheckM v.1.1.3 [58], and taxonomic classification was performed using GTDB-Tk v.1.7.0 [59]. The details have been provided in the Supplementary Materials, Table S1.

### 2.7. In Silico MLST

To determine the sequence types, the study used the MLST tool v2.23.0 [60], which uses the pubMLST website (https://pubmlst.org/; accessed on 13 July 2023) developed by Jolley and Maiden [61] and sited at the University of Oxford. The Wellcome Trust funded the development of that website. The latest *Listeria* ST scheme was obtained from BIGSdb-Lm (https://bigsdb.pasteur.fr/listeria/, accessed on 21 July 2023) [62] and incorporated into the MLST tool.

### 2.8. Resistance and Virulence Profiles

ABRicate [63] detected antimicrobial resistance genes and virulence factors. The application was run with default parameters, and the NCBI database was selected for AMR detection. This database was locally updated on 2 November 2022 and, at the time of usage, included 6334 AMR genes (https://doi.org/10.1128/AAC.00483-19) (accessed on 26 March 2024). The “vfdb” database, updated on 2 November 2022, was used for virulence factors and contained 4332 virulence factors (https://doi.org/10.1093/nar/gkv1239) (accessed on 26 March 2024). The virulence profile is based on the presence/absence of a virulence gene in an isolate. In essence, it is a binary matrix consisting of 0’s and 1’s, with each row representing an isolate and each column a putative virulence gene. Isolates with a similar profile based on the presence or absence of virulence factors will cluster together.

The minimum spanning trees for the virulence factors according to the different industries and sample/food types were constructed based on the presence/absence of a virulence factor. When constructing a minimum spanning tree using a binary matrix as input, no weight was assigned to virulence factors. The LIPI genes were considered individually as being different virulence factors.

### 2.9. Construction of the Phylogenetic Tree for L. monocytogenes Isolates and Correlation with Source and Type of Samples

A core SNP phylogeny was constructed using Snippy v.4.6.0 (https://github.com/tseemann/snippy; accessed on 26 July 2023) and the reference *L. monocytogenes* EGD-e genome (AL591824). FastTree v.2.1.11 [64] was used to infer a phylogenetic tree, visualized in R with ggtree [65].

### 2.10. Provirus/Prophage Detection

GeNomad v.1.5.1 [66] facilitated virus detection by enabling aggressive filtering (“--conservative”) and score calibration (“--enable-sc-calibration”) flags in the “end-to-end” execution mode.

### 2.11. Detection of CRISPR-Cas System

The standalone version of CRISPRCasFinder v.4.3.2 was used to detect CRISPRs and cas genes and classify CRISPR-cas systems [67,68,69].

### 2.12. Data Analysis

The data generated in the current study were analysed using R v.4.3.2 [70], implemented in RStudio v.2023.06.0.421 [71]. Distance matrices were calculated using the “daisy” function with the “gower” parameter specified to determine Gower distances with the R package “cluster” [72]. Minimum spanning trees were calculated using the “ape” package [73], with the “mst” function, and visualized using “igraph” [74] and “ggnetwork” [75] R packages “ggstatsplot” [76], “ggsci” [77], and “ggpubr” [73] were further used for data analysis and visualization. Bar charts were produced using the “ggstatsplot” function “ggbarstats”, and a Chi-squared test for given probabilities was used to test for significant differences at an alpha level of 0.05.

## 3. Results

### 3.1. Frequency of Detection of STs and Genetic Materials

#### 3.1.1. Detection of STs in *L. monocytogenes* According to Industries and Sample/Food Types

Table 1 shows the frequency and distribution of STs in *L. monocytogenes* isolates in the industries. For the 60 isolates of *L. monocytogenes*, the overall frequency of STs and genetic elements whose profiles were investigated was as follows: seven STs were detected at a frequency from 1.7% (ST224) to 46.7% (ST204). For the seven STs detected, three STs (12.9%), five STs (71.4%), and six STs (85.7%) were found in isolates obtained from cattle farms, abattoirs, and retail outlets, respectively, with no statistically significant difference (*p* = 2.0 × 10^−3^).

**Figure 1:**
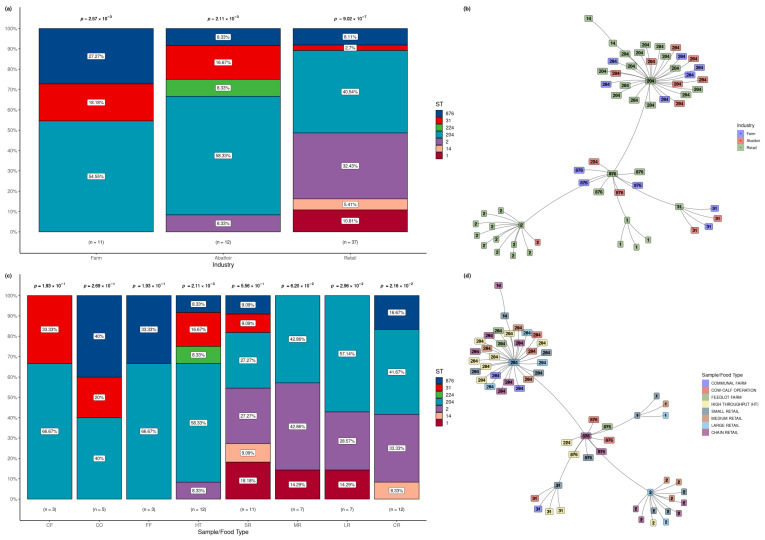
(**a**). Frequency of *L. monocytogenes* sequence types by industry. Significant over-representation of ST204 was found in all three industries, with ST2 additionally found more often than expected in retail. Farms only displayed 3 of the 7 STs detected, followed by abattoirs with 5 STs and the retail industry with 6–7 STs. ST224 was found exclusively in retail samples in abattoirs, ST1, and ST14, with ST2 uniquely shared between the abattoir and retail industries. ST31, ST204, and ST876 were distributed across all the industries. The numbers (1, 14, 2, 204, 224, 31, and 876) represent the STs detected in the isolates. (**b**). Minimum spanning tree based on sequence types for *L. monocytogenes* detected across the different industries. Shared STs across the industries are visible in the multicoloured clusters, with STs unique to particular industries evident based on more homogeneous coloured clusters. The numbers (1, 14, 2, 204, 224, 31, and 876) represent the STs detected in the isolates. (**c**). Frequency of *L. monocytogenes* sequence types by sample/food type. Significant overrepresentation of ST204 was found in High-Throughput (HT), large retail, and chain retail. The numbers (1, 14, 2, 204, 224, 31, and 876) represent the STs detected in the isolates. The letters represent the following: CF, communal farm; CC, cow-calf operation; FF feedlot farm; HT, High-Throughput; SR, small retail; MR, medium retail; LR, large retail; CR, chain retail. (**d**). Minimum spanning tree based on sequence types for *L. monocytogenes* detected across the different industries. Shared STs across the industries are visible in the multicoloured clusters, with STs unique to particular industries evident based on more homogeneous coloured clusters. The numbers (1, 14, 2, 204, 224, 31, and 876) represent the STs detected in the isolates.

Figure 1a shows the frequency distribution of the STs in the isolates of *L. monocytogenes* according to the three industries (cattle farms, cattle abattoirs, and retail outlets). Across the 11 cattle farm isolates, three STs were detected. ST204 was predominantly observed and statistically significant (*p* = 2.7 × 10^−3^) with a higher frequency (54.5%) compared with ST876 (27.3%) and ST31 (18.2%). Among the five STs found in the 12 isolates recovered from the abattoirs, ST204 was predominantly detected at a statistically significant (*p* = 2.11 × 10^−3^) higher frequency (58.3%) compared to the frequency range of 8.3% (ST2, ST224, and ST876) to 16.7% (ST31) in the other four STs. The 37 isolates of *L. monocytogenes* from the retail outlets yielded the highest number of different STs (n = 6), with ST204 detected at the highest frequency (40.5%) and ST31 least detected (2.7%). The differences in the frequency distribution of STs were statistically significant (*p* = 9.02 × 10^−7^).

Overall, ST204 was significantly overrepresented in all three industries, with ST2 found more often than expected in the retail industry. ST224 was found exclusively in abattoirs, ST1, and ST14 were found only in retail samples, and ST2 was uniquely shared between the abattoir and retail industries. ST31, ST204, and ST876 were distributed across all industries.

The minimum spanning tree based on sequence types for *L. monocytogenes* detected across the different industries shows that ST1 and ST14 were only detected in the retail industry (Figure 1b). ST2 was predominant in the retail industry, with only one occurrence in abattoirs. ST31, ST204, and ST876 were spread across all three industries, with ST224 unique to the abattoirs.

For the eight sample/food types analysed, the number of STs detected ranged from two (ST3 and ST204) in communal farm isolates to six (ST1, ST14, ST2, ST204, ST31, and ST876) in small retail samples. Within each sample/food type, the frequency distribution of STs varied significantly (*p* < 5.0 × 10^−2^) (Figure 1c). ST204 was the most predominantly detected, with the highest frequency in all. However, there are unique distributions of some STs. Of relevance is the fact that ST2 was detected in five sample/food types (HT abattoirs and the four types of retail outlets: small, medium, large, and chain), ST224 was found only in HT abattoirs, ST1 was present only in three sample/food types (small, medium and large retail outlets), and ST14 was found only in sample types (small and chain retailers).

The respective sample/food types for each isolate of *L. monocytogenes* are displayed in the MST based on STs detected across the sample/food types in Figure 1d. Clustering based on ST was evident, and the spread across various sample/food types for each ST was interesting. A cluster for ST1 was found for small, medium, and large retail industries, with the ST204 cluster representing all the sample/food types in this study. The ST2 grouping represented sample/food types from all the retail sectors, i.e., small, medium, large, and chain, with one occurrence in the high throughput processing environment. Supplementary data, Table S2, shows the details of the sources, sample types, and STs of the 60 isolates of *L. monocytogenes* across the industries and sample/food types.

#### 3.1.2. Phylogenies of *L. monocytogenes* According to STs, Industry, and Sample/Food Type

**Figure 2:**
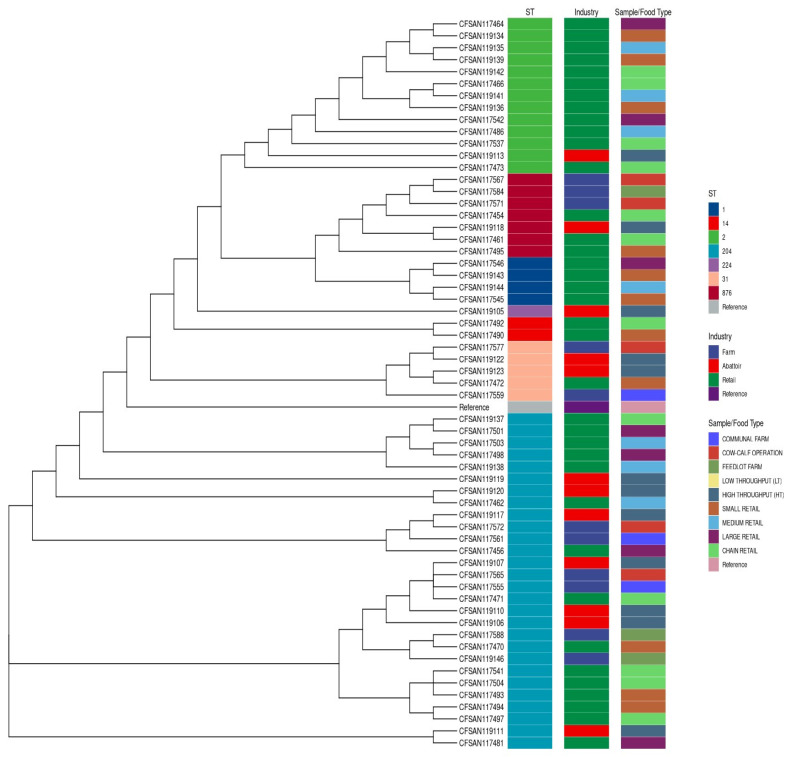
Phylogeny of *L. monocytogenes* was detected across the industries based on SNPs present in all samples (core SNPs). The first colour legend indicates the ST, the second the industry for each isolate, and the third represents the sample/food type. The tree demonstrates the grouping of isolates by ST based on the sequential use of colours in the first colour legend, with the second colour legend displaying an affinity for specific STs within industries. The numbers (1, 14, 2, 204, 224, 31, and 876) represent the STs detected in the isolates.

The phylogenetic tree depicted in Figure 2 indicates relatedness based on ST. The isolates were grouped according to ST and exhibited affinity for certain STs in the different industries. The retail industry displayed a propensity for ST1, ST2, and ST14. The promiscuous nature of ST204 was further highlighted as it was found across all three industries and all sample/food types.

### 3.2. Distribution of Clonal Complexes (CC) among L. monocytogenes Isolates

The 60 isolates of *L. monocytogenes* belonged to six CCs at the following frequencies: three in lineage I (CC1: ST1 and ST876, CC2: ST2, and CC224: ST224), and three in lineage II (CC14: ST14, CC31: ST31, and CC204: ST204). A total of 25 (41.7%) and 35 (58.3%) *L. monocytogenes* were allocated to lineage I and lineage II, respectively. The classification and distribution of the CCs and lineages of *L. monocytogenes,* according to the sources (industries and sample/food types), are shown in the Supplementary Materials, Table S3.

Overall, CCs were detected across cattle farms, abattoirs, and retail outlets at the following frequencies: CC1 (18.3%), CC2 (21.7%), CC224 (1.7%), CC14 (3.3%), CC31 (8.3%), CC204 (46.7%). The differences were statistically significant (*p* < 5.0 × 10^−2^).

Among the 11 isolates of *L. monocytogenes* recovered from cattle farms (communal, cow-calf, and feedlot), only three CCs were detected: CC1 (27.3%, lineage I), CC31 (18.2%), and CC204 (54.5%), all classified in lineage II. The frequency of CCs across the sample types (environment, faeces, and feeds) did not vary significantly (*p* > 5.0 × 10^−2^), but CC1 (27.3%) and CC204 (54.5%) were predominant.

Among the 12 isolates from the abattoirs (HT), five CCs were detected, namely CC1 (8.3%), CC2 (8.3%), and CC224 (8.3%) in lineage I, and CC31 (16.7%) and CC204 (58.3%) in lineage II. The CC204 was detected at a statistically significant (*p* = 6.8 × 10^−3^) higher frequency than other CCs, but the differences in the frequency of CCs across sample types (environment, faeces, and carcass) were not statistically significant (*p* > 0.05).

Five CCs were found among the 37 isolates of *L. monocytogenes* recovered from retail outlets: CC1 (18.9%) and CC2 (32.4%) in lineage I, and CC14 (5.4%), CC31 (2.7%), and CC204 (40.5%) in lineage II. CC2 and CC204 were detected at statistically significant (*p* < 1.0 × 10^−4^) high frequencies compared to the others. However, the sample types (RTEs, milled beef, raw beef, and offal and organs) had no significant (*p* > 5.0 × 10^−2^) effect on the frequency of CCs.

### 3.3. Occurrence of Virulence Factors in L. monocytogenes Isolates According to the Industries and Sample/Food Samples

The virulence factors detected in the *L. monocytogenes* isolates according to their class, five industries, and sample/food type are shown in the Supplementary Materials, Table S4. In the 60 isolates of *L. monocytogenes*, a total of 44 virulence factors, of which six (prfA, *plcA*, *hly*, *mlp*, *actA*, and *plcB*) were LIPI-1 genes, were found. Additionally, eight virulence factors in the LIPI-3 cluster were detected. Also, six internalin family members and other virulence factors that perform important roles in the pathogenesis of listeriosis were detected, such as those responsible for surface protein adherence (n = 1), adherence (n = 4), invasion (n = 6), intracellular survival (n = 3), stress-related (n = 3), and immune modulation (n = 2), among others.

Twenty-six virulence factors were detected in all 60 (100%) *L. monocytogenes* isolates. In comparison, 18 virulence factors were detected at a frequency range of 1.7% (1/60) for *EcbA*/*fss3* to 98.3% (59/60) for *inlA* and *inlP*. The differences were statistically significant (*p* < 1.0 × 10^−3^). However, the differences in the frequency of the virulence genes across the three industries (cattle farms, abattoirs, and retail outlets) were not statistically significant (*p* > 5.0 × 10^−2^).

**Figure 3:**
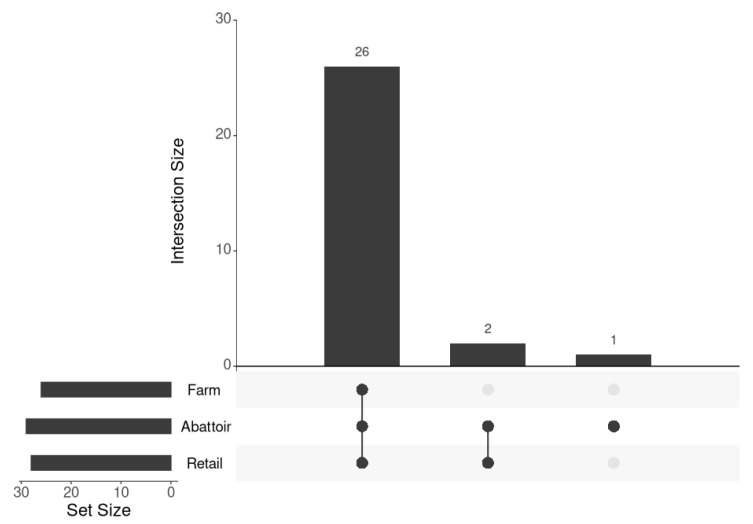
Shared and unique virulence factor (VF) genes across the industries. A total of 44 different VF genes were found in the 60 isolates. Farm isolates had 26 (59.1%; 26/44) VF genes shared by all 11 samples, abattoirs had 29 (65.9%) from 12 isolates, and the retail industry had 28 (63.6%) VF genes found in all 37 samples. Each industry’s core VF gene list was inspected for shared and unique genes between the different industries. The black dots in the bottom right panel indicate the group of interest, with connecting lines representing an intersection between the groups. No lines indicate a unique set within a group.

Shared and unique virulence factors (VF) across the industries are shown in Figure 3 for the 44 virulence factors in all 60 isolates of *L. monocytogenes.* Twenty-six core VF genes were found to be shared across all the industries (*bsh*, *clpC*, *clpE*, *clpP*, *fbpA*, *gtcA*, 419 *hbp1*/*svpA*, *hbp2*, *hly*, *hpt*, *iap*/*cwhA*, *inlB*, *inlC*, *inlK*, *lap*, *lntA*, *lpeA*, *lplA1*, *lspA*, *mpl*, *oatA*, 420 *pdgA*, *plcA*, *plcB*, *prfA*, *prsA2*). The abattoir and retail industries also uniquely shared VF (*inlA*, *inlP*) genes in all the samples from those industries. One VF (*inlF*) was present 422 in all the abattoir samples, but only in some retail and farm samples.

#### Frequency Distribution of Virulence Factors According to the Industries and Sample/Food Types

**Figure 4:**
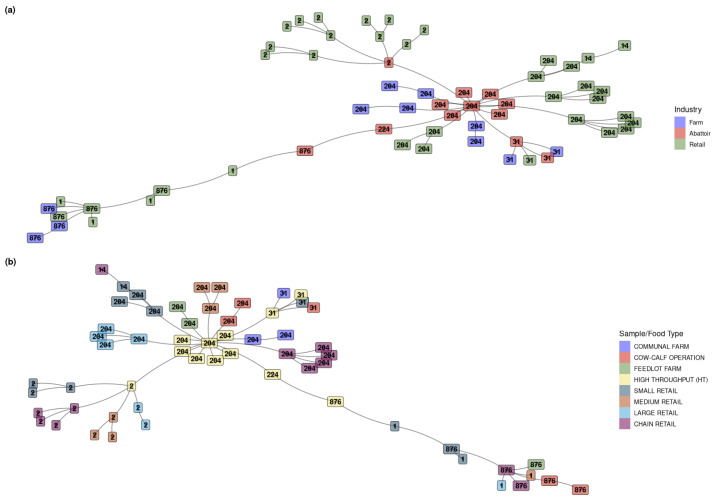
(**a**). Minimum spanning tree based on the presence/absence of virulence factors for *L. monocytogenes* detected across the different industries. Each isolate is represented by sequence type as text and coloured by industry. This is similar to one based on ST, but interest is the cluster on the left. Previously, ST1 and ST876 clustered independently; they clustered together with the virulence factor tree. ST224 is found in the ST876 cluster and is grouped individually in the ST tree. From this, the virulence profiles for ST876 and ST1 are very similar. The numbers (1, 14, 2, 204, 224, 31, and 876) represent the STs detected in the isolates. (**b**). Minimum spanning tree based on the presence/absence of virulence factors for *L. monocytogenes* detected across the sample and food types. Each sample is represented by sequence type as text and coloured by industry. This tree is similar to one based on ST, but the cluster on the left is interesting. Previously, ST1 and ST876 clustered independently, whereas with the virulence factor tree, they clustered together. In the ST tree, ST224 was found within the ST876 cluster, but now it groups individually. From this, the virulence profiles for ST876 and ST1 are very similar.

According to the industries, the clustering of the samples based on virulence gene profiles indicated distinct groupings (Figure 4a). These groups were aligned with the designated ST, and found to be homogeneous according to the ST assignment, except ST224, which was found in a cluster otherwise populated by ST876. ST1 and ST14 were only detected in the retail industry. ST2 was predominant in the retail industry, with only one occurrence in abattoirs. ST31, ST204, and ST876 were spread across all three industries, with ST224 unique to the abattoirs. Overall, the main cluster consisted of 28 isolates, all belonging to ST204, and was represented by all the industries, specifically farms (n = 6), abattoirs (n = 7), and retail (n = 15). The finding that the isolates belonging to the same ST have similar virulence profiles explains why this causes the main cluster.

According to the sample/food types, the MST is similar to one based on ST, but the cluster on the left is interesting (Figure 4b). Previously, ST1 and ST876 clustered independently, whereas with the virulence factor tree, they clustered together. In the ST tree, ST224 was found within the ST876 cluster, but now it groups individually. From this, the virulence profiles for ST876 and ST1 seem very similar.

Overall, the main cluster comprised all 28 ST204 isolates, which originated from all eight sample/food types, and a smaller cluster consisting of only ST2 (n = 13) was distributed across five sample types: HT abattoir (n = 1), large retail outlet (n = 2), medium retail outlet (n = 3), small retail outlet (n = 3), and chain retail outlet (n = 4).

### 3.4. Frequency of Resistance Genes in L. monocytogenes Isolates

All isolates of *L. monocytogenes* contained the AMR genes *fosX* (product—fosfomycin resistance hydrolase *FosX*; phenotype—fosfomycin) and *vga*(G) (product—ABC-F type ribosomal protection protein *vga*(*G*); phenotype—lincosamide). Additional information is provided in the Supplementary Materials, Table S5.

### 3.5. Occurrence of AMR Plasmids in L. monocytogenes Isolates

Only one AMR plasmid, NF033156, was detected in our study at a frequency of 5% in three isolates of *L. monocytogenes* (two from retail outlets and one from an abattoir).

In addition, 48 (80%) isolates were carriers of conjugative plasmids comprising 36 single and 12 mixed plasmids in isolates. Three conjugative plasmids were detected with the following statistically significant (*p* < 1.0 × 10^−3^) different distribution frequencies: MOBP2, 23 (38.3%), MOBV, 10 (16.7%), and FA_orf13; FA_orf17b, 3 (5%). The frequency of detection of the three conjugative plasmids by ST and industry was as follows: MOBP2, ST876 (n = 6, retail outlets: 3, farms: 3), ST204 (n = 1, retail outlet), ST2 (n = 11, retail outlets), ST31 (n = 4, farm: 2, abattoir: 2) and ST1 (n = 1, retail outlet); MOBV, ST2 (n = 10, retail outlets); FA_orf13; FA_orf17b, ST876 (n = 1, retail), ST1 (n = 1, retail) and ST2 (n = 1, abattoir). ST2 from retail outlets was predominant in MOBP2, 47.8% (11/23), and MOBV, 100% (10/10). Details of both types of plasmids are shown in the Supplementary Materials, Table S6.

### 3.6. Frequency of Proviruses/Prophages in the Isolates of L. monocytogenes

Proviruses/Prophages of the class Caudoviricetes were detected in all 60 isolates of *L. monocytogenes* (Supplementary Materials, Table S7).

### 3.7. Frequency of the CRISPR-Cas System in L. monocytogenes Isolates

The detection frequency of the CRISPR-Cas system in *L. monocytogenes* was 10% (6/60), with an even distribution of positive isolate by the industry being 18.2% (2/11), 16.7% (2/12) and 5.4% (2/37) for isolates from cattle farms, abattoirs, and retail outlets (*p* = 3.2 × 10^−1^). In the six samples, a CRISPR-cas subsystem (Class1-Subtype-I-B_1 was detected. The details are shown in the Supplementary Materials, Table S8.

### 3.8. Provirus/Phage and AMR Co-Location (Provirus or Phage as Classification)

**Figure 5:**
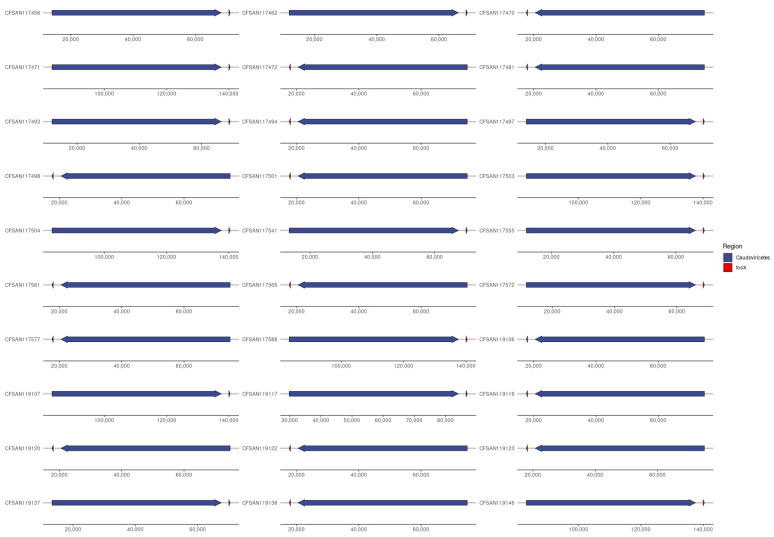
Provirus/phage and AMR co-location (provirus or phage as classification).

Proviruses/Prophages of the class Caudoviricetes (Viruses; Duplodnaviria; Heunggongvirae; Uroviricota; Caudoviricetes) were detected in all 60 isolates of *L. monocytogenes*. In 30 of the isolates (Figure 5), the provirus/prophage was detected on the same contig as the AMR gene *fosX* (FOSFOMYCIN), and always within 2500 bp of each other, first with the provirus/prophage and then the *fosX* (strand-specific). This was found across all three industries (farm = 7, abattoir = 7, retail = 16) and only in ST31 (four out of the five total in the data) and ST204 (26 out of the 28 total in data). From this, the provirus/prophage is the vector for the *fosX* gene in specific STs, particularly ST31 and ST204. The gene space around the *fosX* gene was conserved in all 30 isolates with engB (GTP-binding protein EngB) always between the phage and *fosX*, followed by bdlA (biofilm dispersion protein BdlA), rimJ (ribosomal-protein-S5-alanine N-acetyltransferase), zitB (zinc transporter ZitB), mprF (phosphatidylglycrol lysyltransferase), Epimerase family protein and recX (regulatory protein RecX).

### 3.9. Characteristics of L. monocytogenes Recovered from RTE Beef Products

Seven isolates of *L. monocytogenes* were recovered from RTE products comprising Vienna (n = 1), ‘biltong’ (n = 1), and beef ‘polony’ sampled from the four categories of retailers (chain, large, medium, and small) (Table 2). Isolates of *L. monocytogenes* of serogroup 11a and ST204 were detected only in beef polony, while ST876 and ST2 were detected in Vienna and ‘biltong’, respectively. Carriage of virulence factors was high, ranging from 32 for biltong and polony isolates to 39 for beef polony isolates of the 44 virulence factors detected in the study. Clonal complexes 1 and 2 were detected in four of the seven RTE products. The six RTE isolates were positive for AMR genes [*vga* (G)] and proviruses/prophages of the class Duplodnaviria, but were all negative for AMR plasmids and the CRISPR-Cas system.

## 4. Discussion

In the most recent outbreak of *L. monocytogenes* in South Africa, considered the largest in the world, *L. monocytogenes* ST6 was determined to be responsible. It was due to consuming contaminated ‘polony’, an RTE product [6]. The epidemiology, WGS analysis, and the comparison of South Africa’s outbreak with reports from other countries have been documented [24,80,81]. Beef and beef-based products have been reported to be vehicles for listeriosis in different countries [3,82]. Following the outbreak in the country, WGS analyses have been used to investigate the population structure of *L. monocytogenes* isolated in the meat value chain in South Africa [40,53]. However, the current study is the first to use WGS and bioinformatics analyses to characterize *L. monocytogenes* recovered from the country’s beef production chain (cattle farms, abattoirs, retail outlets).

Of the seven STs detected in our study, three were found at comparatively high frequencies for CC1: ST876 (11.7%), CC2: ST2 (21.7%), and CC204: ST204 (46.7%). In comparison, a study of 217 *L. monocytogenes* isolates recovered from red meat and poultry value chain in South Africa [53] reported 20 STs, comprising ST204 (14.7%), ST2 (13.8%), ST1 (11.5%), ST9 (11.1%), and ST321 (9.7%). It is pertinent to mention that the current study and two other studies, one conducted on the food chain [40] and the other on meat and meat products [55], all of which were after the large human listeriosis outbreak of 2017–2018 [6], failed to detect ST6 of *L. monocytogenes*, which was responsible for the outbreak. However, it cannot be over-emphasized that ST204, the most frequently detected ST in the three studies, may pose a potential food safety concern since it has been associated with human listeriosis elsewhere [21,83,84]. It has been documented that ST204 is predominant in meat products in Australia and France [85,86] and in food processing plants [87]. Furthermore, other STs observed in our study have been detected in meat and meat products and other foods implicated in cases and outbreaks of listeriosis [25,87,88].

The STs/clonal complexes (CC) and lineage information have been used to predict the virulence or pathogenicity potential of *L. monocytogenes* recovered from human cases and foods [26,89,90]. This association of CCs and lineages of *L. monocytogenes* with virulence is partially linked to the type and number of virulence factors they carry. It is, therefore, significant that *L. monocytogenes* isolates allocated to CC1 and CC2 in lineage I were detected in 24 (40%) of the isolates. They are frequently involved in human listeriosis, and were considered hypervirulent clones [26,91,92,93], and CC204: ST204 in lineage II constituted 46.7% of our isolates, and has been documented to be isolated mostly from foods and recovered from human listeriosis in South Africa [40,53] and elsewhere [94,95]. In our study, all 60 isolates were carriers of 5 LIPI-1 virulence factors (*prfA*, *plcA*, *hly*, *mpl*, and *plcB*) and 8 LIPI-3 cluster virulence factors (*IlsA*, *IlsB*, *llsD*, *IlsG*, *IlsH*, *IlsP*, *IlsX*, and *IlsY*) were detected in 13.3–20% of our isolates, which have been demonstrated to play an important role in the virulence/pathogenicity of *L. monocytogenes* [26,96,97].

The distribution of STs of *L. monocytogenes* within and across the three industries was significantly different. However, any association between industries and STs may be limited to the current sampling scope, including the locations and sampling span. STs/CCs of *L. monocytogenes* are known to be frequently introduced and transmitted; therefore, a cross-sectional or ‘snapshot’ study, like ours, will be unable to infer the persistence of the CCs in that location over multiple years. Some STs (ST31, ST204, and ST876) were distributed across all the industries. At the same time, ST224 was found exclusively in abattoir isolates, ST1 and ST14 were detected only in the isolates from the retail industry, and ST2 was shared between the abattoir and retail industries. The differences in the number and types of STs recovered in the three industries may be partly explained by the number of isolates tested per industry, 11, 12, and 37, respectively. Furthermore, the variation in the number of sources could have contributed to the recovery of isolates of *L. monocytogenes* (cattle farms versus abattoirs versus retail outlets). It was also significant that ST204 was predominantly detected in three industries. Other reports have documented differences in the number and frequency of STs in *L. monocytogenes* from these industries by others [98,99]. ST204 was detected at the highest frequency across all the sample/food types tested in other studies [40,53]. Therefore, there is a possibility that ST204 is widespread among *L. monocytogenes* isolates in Gauteng province.

Notably, the LIPI-3 genes were detected at frequencies ranging from 13.3% (*IlsP*) to 20% (*IlsA*, *IlsB*, *IlsG*, *IlsH*, *IlsX*, and *IlsY*) of our isolates. This is because the LIPI-3 gene cluster is involved in the ability of *L. monocytogenes* to infect host cells and survive in the food processing environment [27,28]. These virulence factors perform different roles and functions, such as being responsible for surface protein anchoring, adherence, invasion, immune modulation, and intracellular survival, among others; some virulence factors have been implicated in human listeriosis [3,29,100,101,102]. Equally relevant is the finding that 26 (59.1%) of our 60 isolates shared important virulence factors, including virulence factors belonging to LIPI-1 and LIPI-3 genes. Matle et al. [103] similarly reported the presence of 47 similar virulence factors in six sequenced isolates of *L. monocytogenes* from RTE products in the country.

The current study’s detection of 44 putative virulence factors is considerably lower than the 68 putative virulence factors detected in *L. monocytogenes* in a previous study in South Africa [40]. The differences may be accounted for partly by the origins and types of the sample sources and different *L. monocytogenes* populations resident in these sources. In our study, the *L. monocytogenes* isolates originated from cattle farms (faeces; feeds: grain, grass, and silage), cattle abattoirs (pre-evisceration and post-evisceration carcass swabs, chilled carcass swabs, and effluent), and from retail outlets (raw beef, offal and organs, milled beef, and RTE) in Gauteng province. In contrast, the *L. monocytogenes* isolates analysed by Mafuna et al. [40] originated from raw meat, processed meat, RTE meat products from retailers, and environmental samples obtained from a pig farm during the human listeriosis outbreak investigation to identify the source of the raw materials used to prepare the ‘polony’ [53].

The clustering of virulence factors within and across the three beef industries and sample/food type, as well as the MST based on their STs, is not surprising, but indicates that these variables affect the consumer’s exposure potential to virulent isolates of *L. monocytogenes* in agreement with published reports [90,104]. Our finding of food safety and therapeutic importance is that seven of our isolates of *L. monocytogenes* from RTE beef products were carriers of potentially virulent *L. monocytogenes* STs also harbouring AMR genes. RTE products have been documented to be implicated in most human listeriosis cases or outbreaks [3,12]. Relevant to South Africa is that ‘polony’, one of the three RTE beef products (Vienna, ‘polony’, ‘biltong’) sampled, is a popular delicacy implicated in the recent large outbreak of human listeriosis [6]. It is, therefore, a concern that ‘polony’ constituted five of the seven RTE products where *L. monocytogenes* isolates carried 32–39 virulence factors. Matle et al. [103] detected 142 virulence genes across the sequences of six RTE isolates, which are considerably higher than found in the RTE products in the current study. Other reports have documented the contamination of RTE meat products with *L. monocytogenes* and characterized the isolates regarding their virulence factors and resistance genes [3,93,105,106].

Only two AMR genes, *fosX*, and *vga*(*G*), which encode phenotypic resistance to fosfomycin and lincosamide, respectively, were detected in our study in all 60 isolates. This is not a surprise because, in South Africa, some antimicrobial agents, including fosfomycin, tetracycline, and sulphonamides, are legally allowed to be sold over-the-counter for use in the livestock industry. Therefore, these farmers use antimicrobial agents to treat livestock and as growth promoters in the livestock industry [107,108,109] without veterinary oversight. Thus, our study’s exceptionally high detection frequency of the *fosX* gene (100%) may have therapeutic implications. In agreement with the current study was the detection of the *fosX* gene in all (100%) *L. monocytogenes* isolates recovered from RTE products of animal origin (100%) [96] and from the food chain [40] in South Africa. However, unlike our current study, where only *fosX* and *vga*(*G*) were detected, Mafuna et al. [40] found four AMR genes (*fosX*, *lin*, *norB*, and *mprF*) in all isolates of *L. monocytogenes* obtained from the country’s meat food chain. The origin of the samples and isolate genotypes in both studies may account for the differences in their findings. Additionally, it is known that different genotypes can have different AMR gene profiles [110]. The detection of the AMR gene, *fosX*, in all (100%) *L. monocytogenes* isolates in the current study may pose therapeutic implications in infected humans should it be expressed. Published reports of studies elsewhere have documented a similarly high frequency of *fosX* gene carriage in *L. monocytogenes* isolated from RTE products, such as the 100% frequency detected in Chile by Parra-Flores et al. [50] and the 97.8% reported in the USA [111].

Only one AMR plasmid, NF033156, was found at a low frequency of 5% (3/60) in our study, and all were from ST204 isolates recovered from an abattoir and two from retail outlets. Matle et al. [103] found no plasmid in a study on six isolates of *L. monocytogenes* recovered from RTE meat products. Of relevance is that Mafuna et al. [40] detected plasmids in 71% of the 143 isolates of *L. monocytogenes* studied, and their detection was ST-specific. Although there were differences in the types and frequencies of AMR plasmids identified in both studies, the over-representation of the plasmids in some STs is common. The differences in the types and frequencies of plasmids recovered from *L. monocytogenes* in both studies may be due to the origins of the samples that yielded the pathogen and the losses of plasmids. Plasmids are essential in the carriage of AMR genes and other genetic materials in *L. monocytogenes* and other bacteria [112,113].

Three conjugative plasmids were detected in 80% of the 60 isolates of *L. monocytogenes* tested in our study, with their detection at statistically significant different frequencies for MOBP2 (38.3%), MOBV (16.7%), and FA_orf13; FA_orf17b (5%). Notably, the occurrence of the plasmids was associated with the STs and the industry; both MOBV and MOBP2 were associated with the STs and the beef retail industries. In agreement with our findings, Mao et al. [114] demonstrated that a conjugative plasmid, pLM1686, was associated with four STs (ST87, ST59, ST9, and ST120) in China. The authors also reported that the plasmid was detected in various isolates of *L. monocytogenes*, and has a self-transmissible ability, providing *L. monocytogenes* the advantage of surviving in adverse environments.

In our study, all 60 isolates were carriers of proviruses/prophages. This is higher than the 90.9% (30/143) found in *L. monocytogenes* previously isolated from the food chain in the country [40]. Proviruses/prophages play critical roles in *L. monocytogenes*, including mediating defence against phage infection, bacterial survival, and persistence in stressful environments [41,42]. Interestingly, proviruses (prophages) in the class Caudoviricetes were detected in all 60 isolates of *L. monocytogenes* isolates in our study, their co-location with AMR gene (*fosX*), and being ST-specific (ST31 and ST204), indicate that the provirus/prophages may serve as the vector for the *fosX* gene.

We detected the CRISPR-Cas subsystem (Class1-Subtype-I-B_1) in 10% (6/60) of the *L. monocytogenes* isolates, which were evenly distributed across the three industries but were highly represented in ST31, 83.3% (5/6). Parra-Flores et al. [50] recovered the CRISPR-Cas system from 71% of RTE foods sampled in Chile. In South Africa, Mafuna et al. [47] detected three CRISPR-Cas system types (CAS-Type IIA system, CAS-Type IB system, and CAS-Type IIC system) in 41 non-pathogenic *Listeria* spp. recovered from meat and food processing facilities (FPF). Regardless of the *Listeria* spp., the CRISPR-Cas system is known to degrade foreign genetic elements, act as an adaptive immune system, and has been documented to help invade the host immune system and has been documented in *L. monocytogenes* isolates [46,114,115,116].

## 5. Conclusions

Our study, which used WGS and bioinformatic analyses of *L. monocytogenes* isolated from the beef production chain comprising three industries (cattle farms, beef abattoirs, and retail outlets) and different types of samples and foods across Gauteng province, provided vital data on the pathogen. The study characterizes the *L. monocytogenes* regarding their STs, carriage of virulence factors, resistance genes, AMR plasmids, proviruses/prophages, and CRISPR-Cas systems, all factors that play some roles in the organism’s pathogenicity. Based on the high frequency of CC1 and CC2 *L. monocytogenes,* most carrying LIPI-1 (73.3–100%) and LIPI-3 (18.3–20%) virulence factors provide evidence that they could pose a food safety risk to consumers. The predominance of these factors (STs, CCs, and virulence factors) in RTE beef products further supports the risk posed by ‘polony’, which was the vehicle of *L. monocytogenes* in the recent large listeriosis outbreak in South Africa. The use of MST and phylogenies revealed clustering of the putative virulence factors according to the isolates’ source and sample/food types, thus providing their relative risk of exposure. *FosX* was detected in all 60 isolates of *L. monocytogenes*, and the high frequency may be due to the over-the-counter availability and unsupervised use of fosfomycin encoded by the gene in the country’s livestock industry. This AMR gene could, therefore, have therapeutic implications if expressed. Finally, the detection of pathogenic STs, CC1, and CC2, and putative virulence factors that have been associated with human listeriosis, the occurrence of AMR genes, plasmids, proviruses/prophages, and CRISPR-Cas system in our isolates recorded from the three industries is the first time genomics was conducted on this kind of dataset in the country, and this gives insights into health implications of *Listeria*.

## Figures and Tables

**Table 1:** Characteristics of *L. monocytogenes* isolates from the three industries (farm, abattoir, and retail) according to the STs and AMR genes.

	No. of Isolates	No. (%) of *L. monocytogenes* Belonging to:						
Industry	Tested	ST1	ST2	ST14	ST31	ST204	ST224	ST876
Cattle farms	11 ^a^	0 (0.0)	0 (0.0)	0 (0.0)	2 (18.2)	6 (54.5)	0 (0.0)	3 (27.3)
Abattoirs	12 ^a^	0 (0.0)	1 (8.3)	0 (0.0)	2 (16.7)	7 (58.3)	1 (8.3)	1 (8.3)
Retail outlets ^c^	37 ^b^	4 (10.8)	12 (32.4)	2 (5.4)	1 (2.7)	15 (40.5)	0 (0.0)	3 (8.1)
*p*-value		0.56	0.044	1	0.134	0.475	1	0.204
Total	60 ^c^	4 (6.7)	13 (21.7)	2 (3.3)	5 (8.3)	28 (46.7)	1 (1.7)	7 (11.7)

^a^ Originated from 23 cattle farms [78], ^b^ obtained from eight abattoirs [78], ^c^ isolates were recovered from 48 retail outlets [79], ^c^ all 60 isolates of *L. monocytogenes* from the three industries were positive for AMR genes (*fosX* and *vga*(*G*)).

**Table 2:** Occurrence of serogroups, STs, virulence factors, clonal complexes, AMR genes, plasmids, AMR plasmid CRISPR-Cas, and proviruses in *L. monocytogenes* recovered from RTE beef products.

	Characteristics of Seven *L. monocytogenes* Isolates Recovered from RTE Products:						
Isolate ID#	CFSAN117454	CFSAN117466	CFSAN117473	CFSAN117497	CFSAN117498	CFSAN117503	CFSAN117545
Source	Chain-Retail	Chain-Retail	Chain-Retail	Chain-Retail	Large-Retail	Medium-Retail	Small-Retail
Sample type	Vienna	Biltong ^a^	Beef polony ^b^	Beef polony ^b^	Beef polony ^b^	Beef polony ^b^	Beef polony ^b^
Serogroup	Ivb	Ivb	IVb	11a	11a	11a	IVb
MLST	876	2	2	204	204	204	1
Clonal complex	1	2	2	204	204	204	1
No. of virulence factors	39	32	32	35	34	34	39
LIPI-1	Positive ^c^	Positive	Positive	Positive	Positive	Positive ^d^	Positive ^e^
Internalins A&B	Positive	Positive	Positive	Positive	Positive	Positive	Positive
LIPI-3	Positive	Negative	Negative	Negative	Negative	Negative	Negative
Others	Positive	Positive	Positive	Positive	Positive	Positive	Positive
AMR gene: *FosX*	Positive	Positive	Positive	Positive	Positive	Positive	Positive
AMR gene: *vga*(*G*)	Positive	Positive	Positive	Positive	Positive	Positive	Positive
AMR Plasmid	Negative	Negative	Negative	Negative	Negative	Negative	Negative
CRISPR-Cas	Negative	Negative	Negative	Negative	Negative	Negative	Negative
Proviruses/prophages	Positive	Positive	Positive	Positive	Positive	Positive	Positive

^a^ Biltong: a delicacy made of spiced dried raw meat (beef and game) widely consumed in the country. ^b^ Beef polony: a popularly consumed product responsible for the 2018–2019 large outbreak of human listeriosis in South Africa. ^c^ Of the six LIPI-1 virulence factors, negative for the *actA* gene. ^d^ Of the six LIPI-1 virulence factors, negative for the *hly* gene. ^e^ Of the six LIPI-1 virulence factors, negative for the *actA* gene.

## Data Availability

All samples have been deposited under NCBI BioProject PRJNA215355 and can be searched based on the isolated CFSAN identifier.

## Supplementary Materials

The following supporting information can be downloaded at: https://www.mdpi.com/article/10.3390/microorganisms12051003/s1, Supplementary Table S1. Coverage of the sequences used for further analysis; Supplementary Table S2. Sample Information: Source and Type of sample, ST and CC of isolates; Supplementary Table S3. Distribution of clonal complexes and lineages in 60 *L. monocytogenes* recovered from cattle farms, abattoirs, and retail outlets in Gauteng province; Supplementary Table S4. Virulence factors; Supplementary Table S5. AMR data; Supplementary Table S6. AMR and Conjugative plasmid genes; Supplementary Table S7. Provirus; Supplementary Table S8. CRISPR; Supplementary Table S9. Type and frequency of virulence factors detected according to the industries. References [29,101,117] are cited in the Supplementary Materials.
